# Supplementary material for: Developmental mitochondrial complex I activity determines lifespan
Source: EMBO Rep. 2025 Mar 17;26(8):1957–83. doi: 10.1038/s44319-025-00416-6 (PMC12019323; doi:10.1038/s44319-025-00416-6)
Supplement: Supplementary file 1 — Appendix [file 44319_2025_416_MOESM1_ESM.pdf]

Appendix Information

Table of Contents

Appendix Figures

Appendix Figure S1: Related to Figure 1……………………………………..………..…..2

Appendix Figure S2: Related to Figure EV1………………………………………….……3

Appendix Figure S3: Related to Figure 2 ……………………………………...….….……4

Appendix Figure S4: Related to Figure EV2…………………………………………..….5

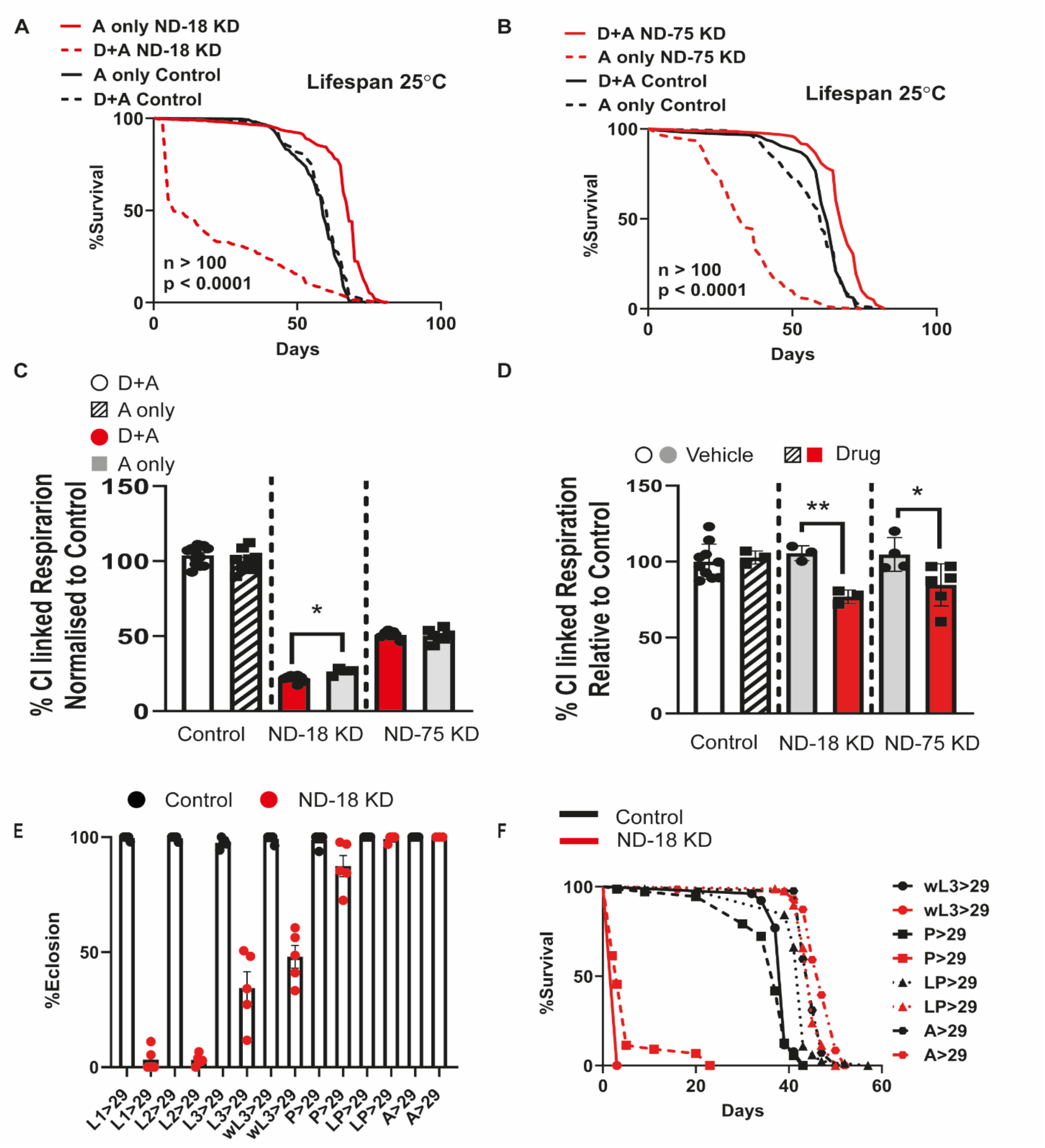

Appendix Figure S1. (A) Survival of control or male flies where CI subunit, ND-18, has been depleted from early development (D+A) or from adulthood (A-only). (B) Survival of control or male flies where CI subunit, ND-75, has been depleted from early development (D+A) or from adulthood (A-only). (C) Normalised levels of CI-linked respiration in controls, ND-18 KD and ND-75 KD 5–7 day adult males. (D) Normalised levels of CI-linked respiration in controls, ND-18 KD or ND-75 KD male 3rd Instar larvae in the presence (drug) or absence (vehicle) of the GS inducer, RU-486. (E) % eclosion of control or flies where depletion of CI subunit, ND-18, has been induced at distinct developmental stages. (F) Survival of controls or male flies where depletion of CI subunit, ND-18, has been induced at distinct developmental stages. *p < 0.05, **p < 0.01, ***p < 0.001.

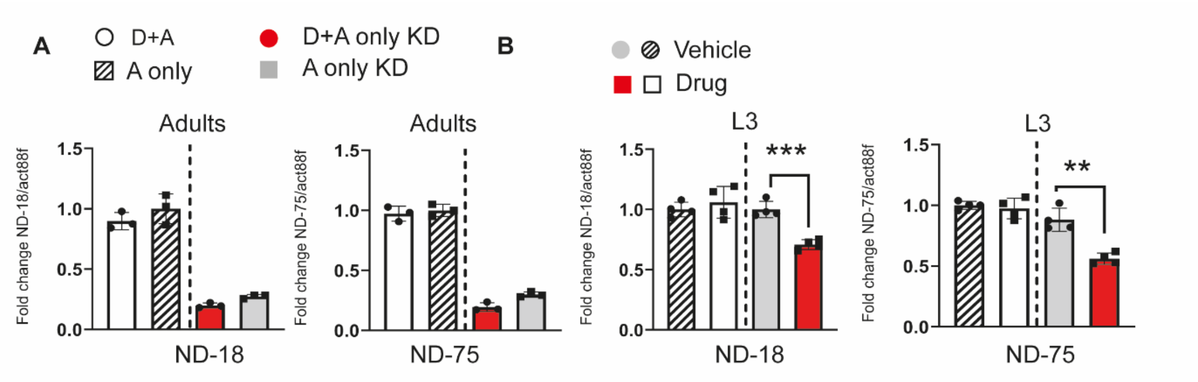

Appendix Figure S2. (A) mRNA quantification of KD in controls and ND-18 KD and ND-75 KD adult males. (B) mRNA quantification of KD in controls and ND-18 KD and ND-75 KD 3rd instar larvae in the presence (drug) or absence (vehicle) of the GS inducer, RU-486. **p < 0.01, ***p < 0.001.

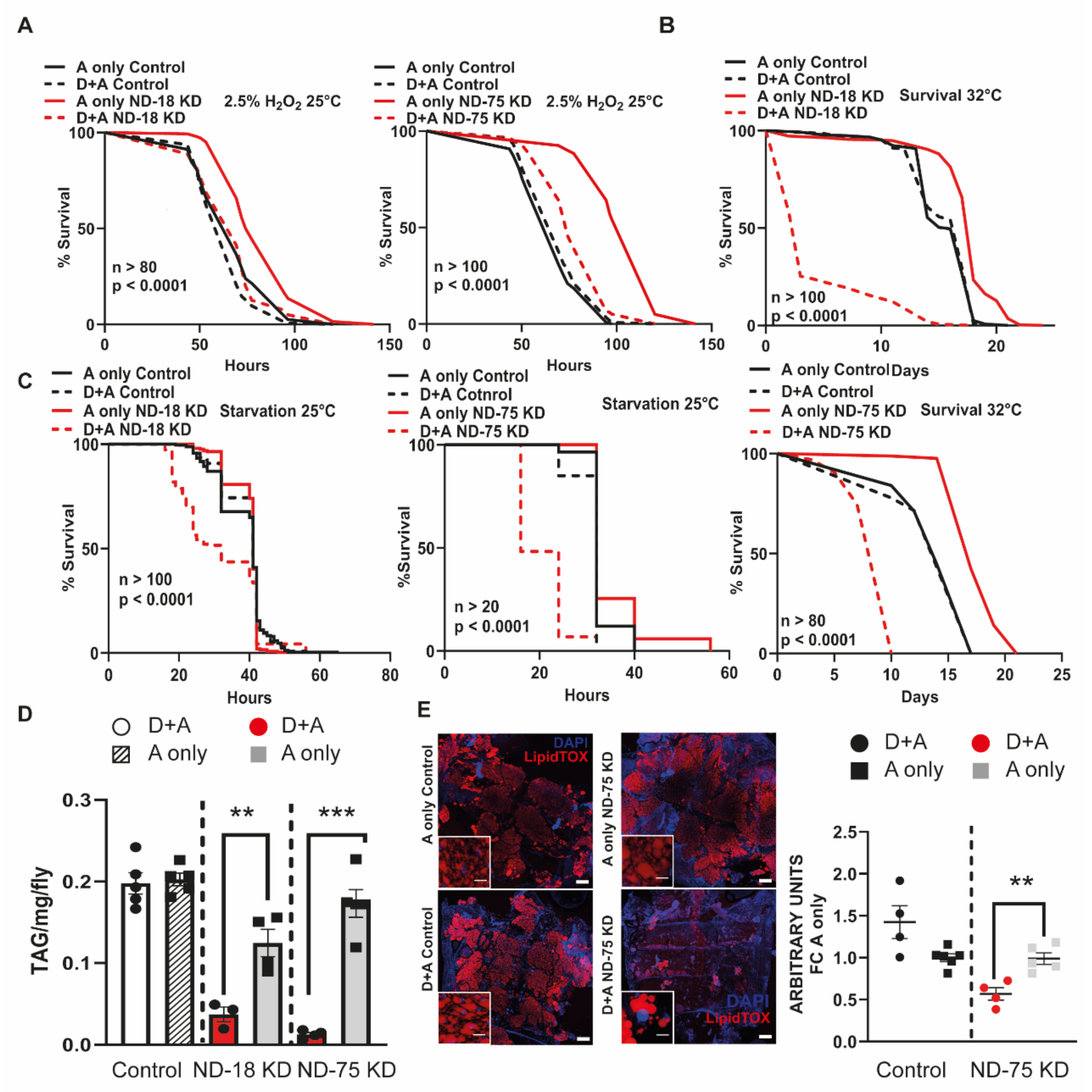

Appendix Figure S3. (A) Survival under oxidative stress conditions of control and male flies where either ND-18 (left) or ND-75 (right) has been depleted from early development (D+A) or from adulthood (A-only). (B) Survival under thermal stress conditions of controls and male flies where either ND-18 (upper) or ND-75 (lower) has been depleted from early development (D+A) or from adulthood (A-only). (C) Survival under starvation conditions of controls and male flies where either ND-18 (left) or ND-75 (right) has been depleted from early development (D+A) or from adulthood (A-only). (D) Quantification of triacylglyceride levels in controls and male flies where either ND-18 or ND-75 has been depleted from early development (D+A) or from adulthood (A-only). (E) Confocal imaging of fat bodies from control and male flies where ND-75 have been depleted from early development (D+A) or from adulthood (A-only) stained with LipidTOX Red and DAPI, scale bar (90 μM), inset scale bar (10 μM). Quantification is displayed on the right. $^{**}p < 0.01$, $^{***}p < 0.001$.

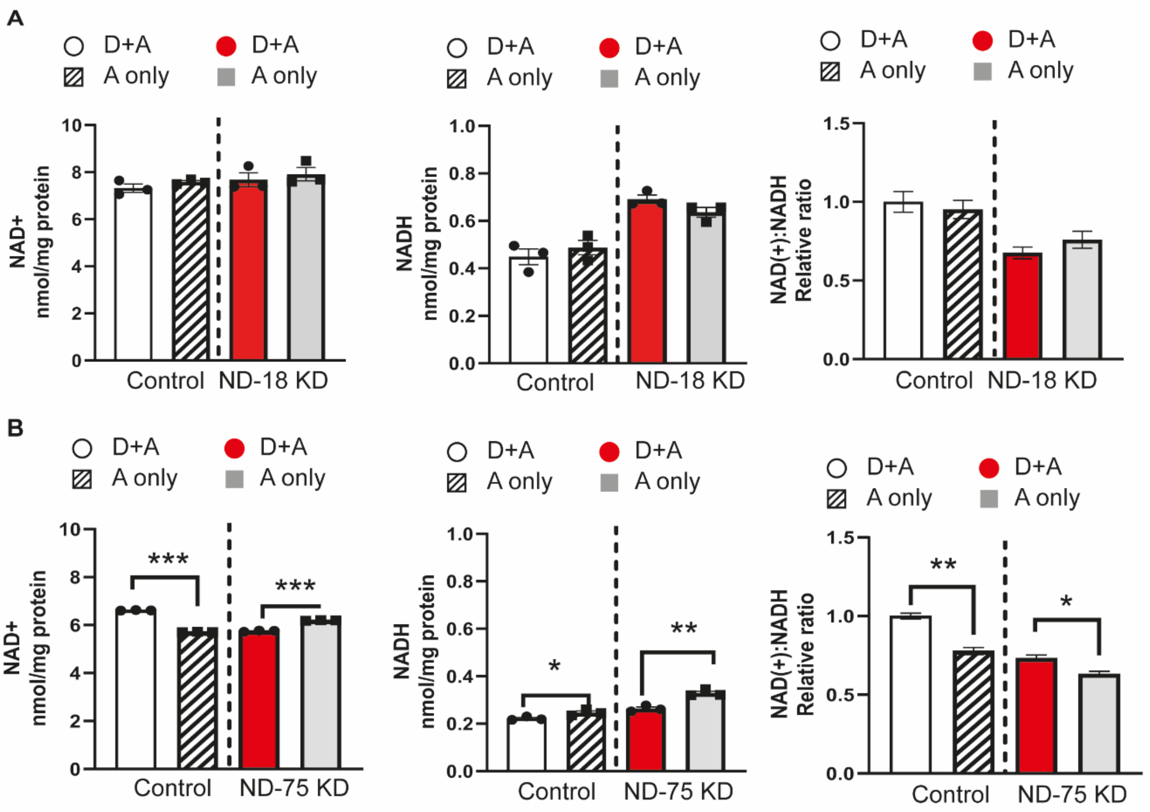

Appendix Figure S4. (A) Levels of NAD+, NADH, and the relative ratio of NAD(+):NADH in controls and ND-18 KD flies. (B) Levels of NAD+, NADH, and the relative ratio of NAD(+):NADH in controls and ND-75 KD flies. *$p < 0.05$, **$p < 0.01$, ***$p < 0.001$.
