# Supplementary figures and images for: Developmental mitochondrial complex I activity determines lifespan
Source: EMBO Rep. 2025 Mar 17;26(8):1957–83. doi: 10.1038/s44319-025-00416-6 (PMC12019323; doi:10.1038/s44319-025-00416-6)

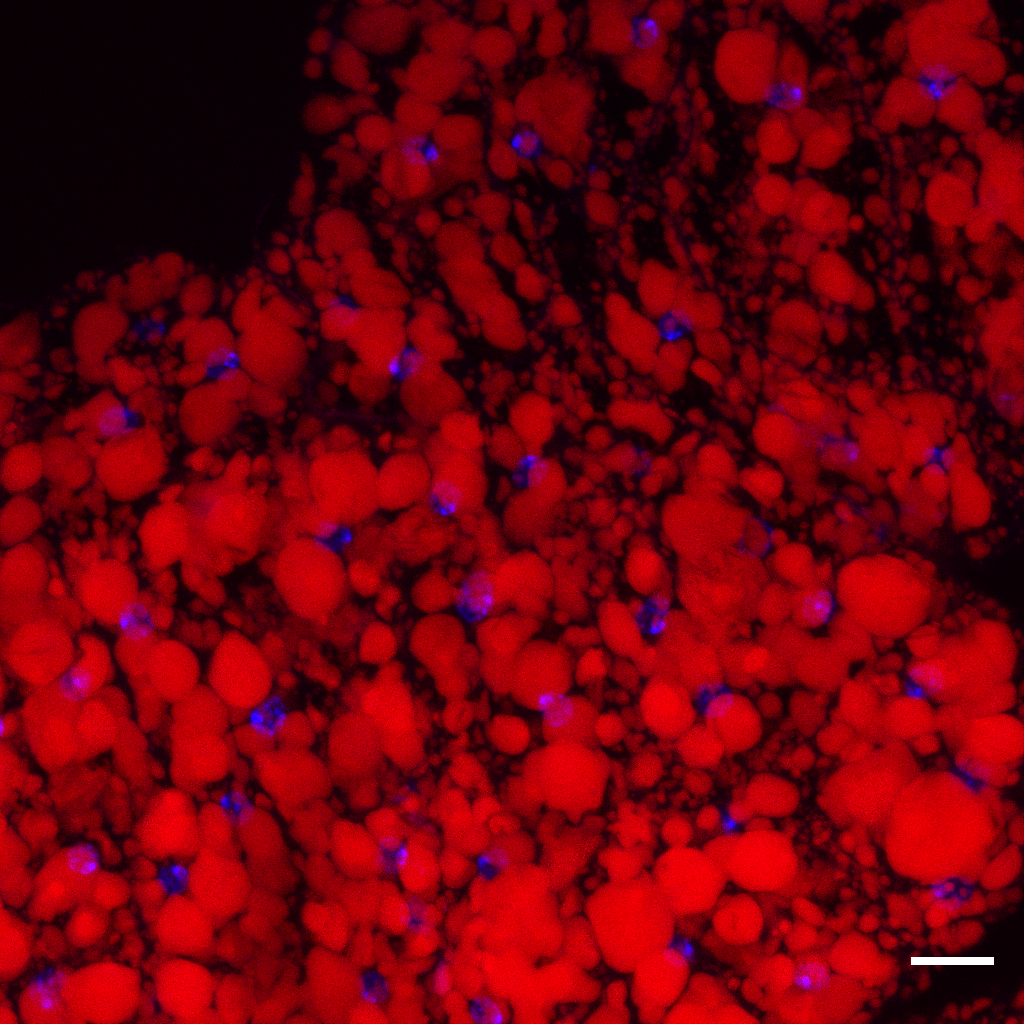

Supplement: Supplementary file 4 — Source data Fig. 2 [file 44319_2025_416_MOESM4_ESM.zip › 2E/MAX_011119.lif - 75MA-FB-63x-2 (RGB).tif]

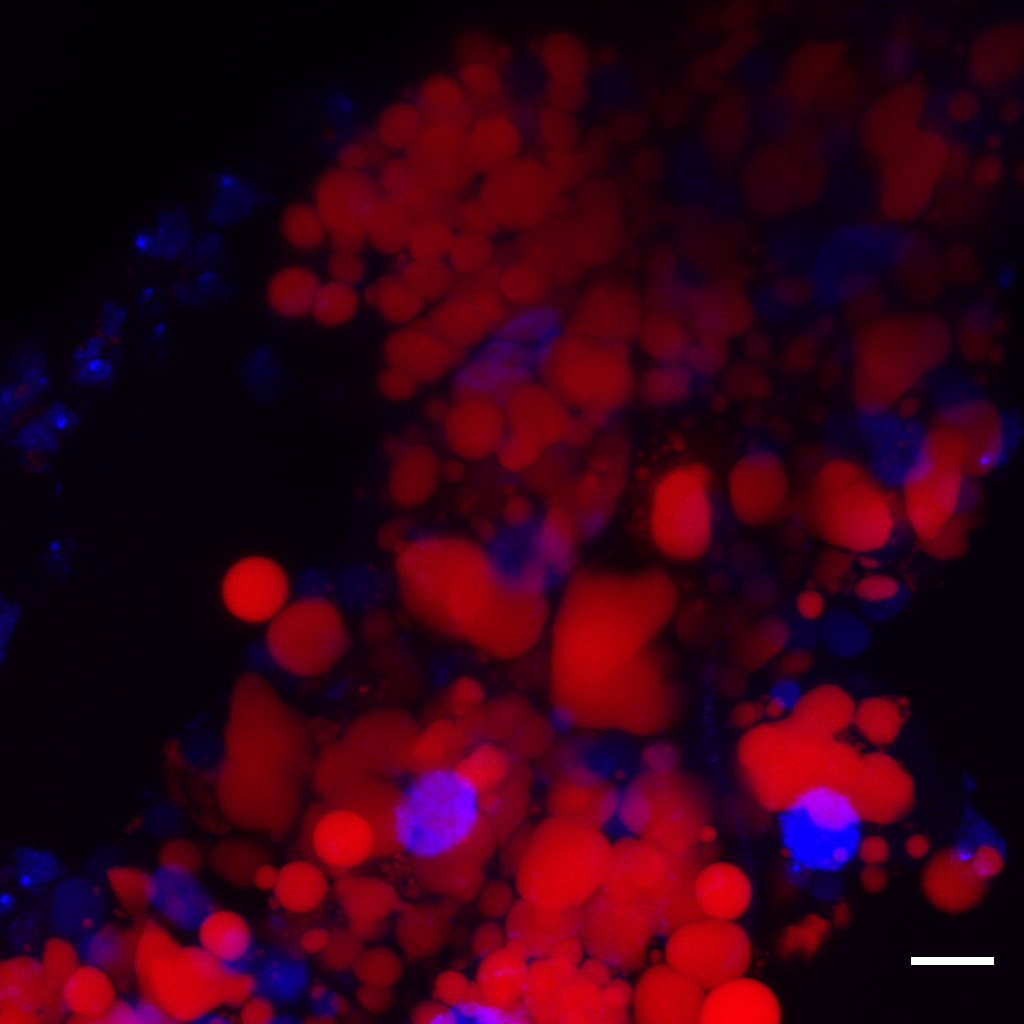

Supplement: Supplementary file 4 — Source data Fig. 2 [file 44319_2025_416_MOESM4_ESM.zip › 2E/MAX_011119.lif - 7MD1A-FB-63x-2 (RGB).tif]

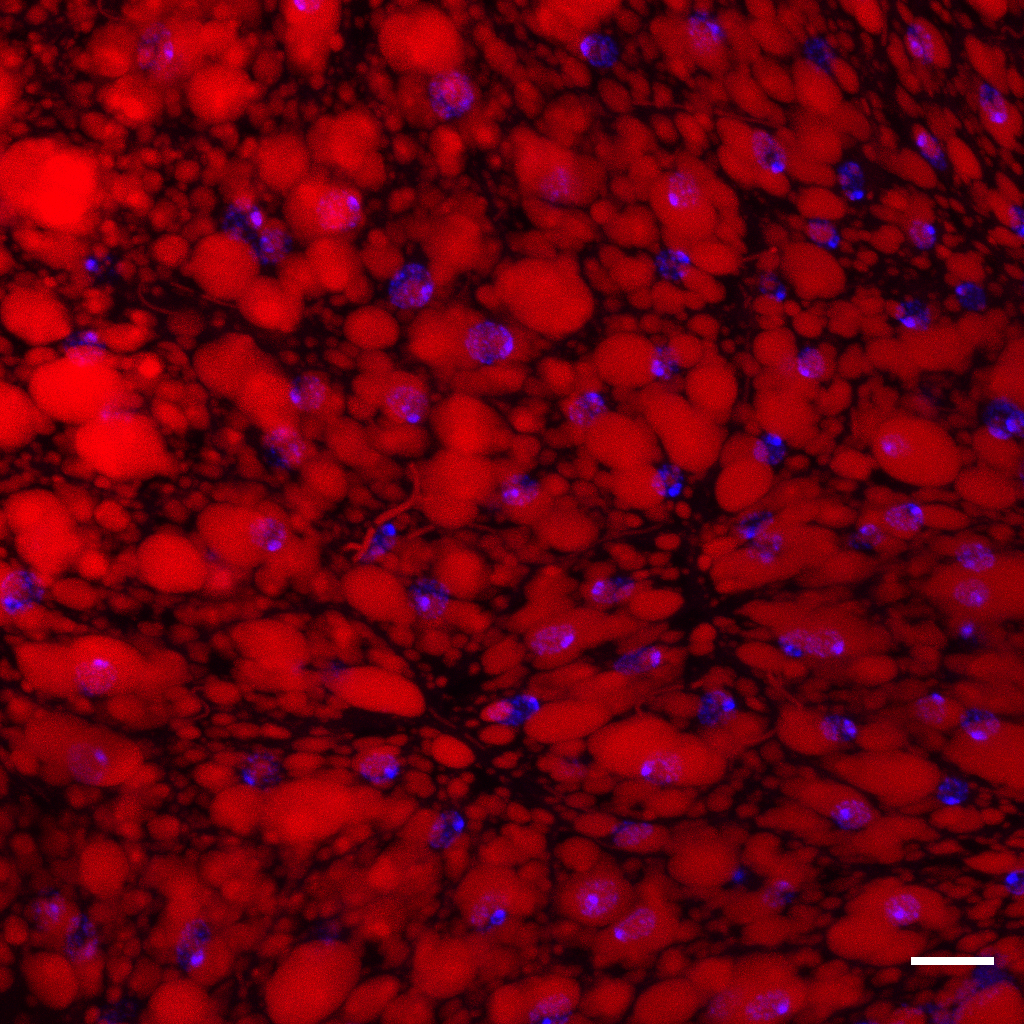

Supplement: Supplementary file 4 — Source data Fig. 2 [file 44319_2025_416_MOESM4_ESM.zip › 2E/MAX_011119.lif - CMD1A-FB-63x-1 (RGB).tif]

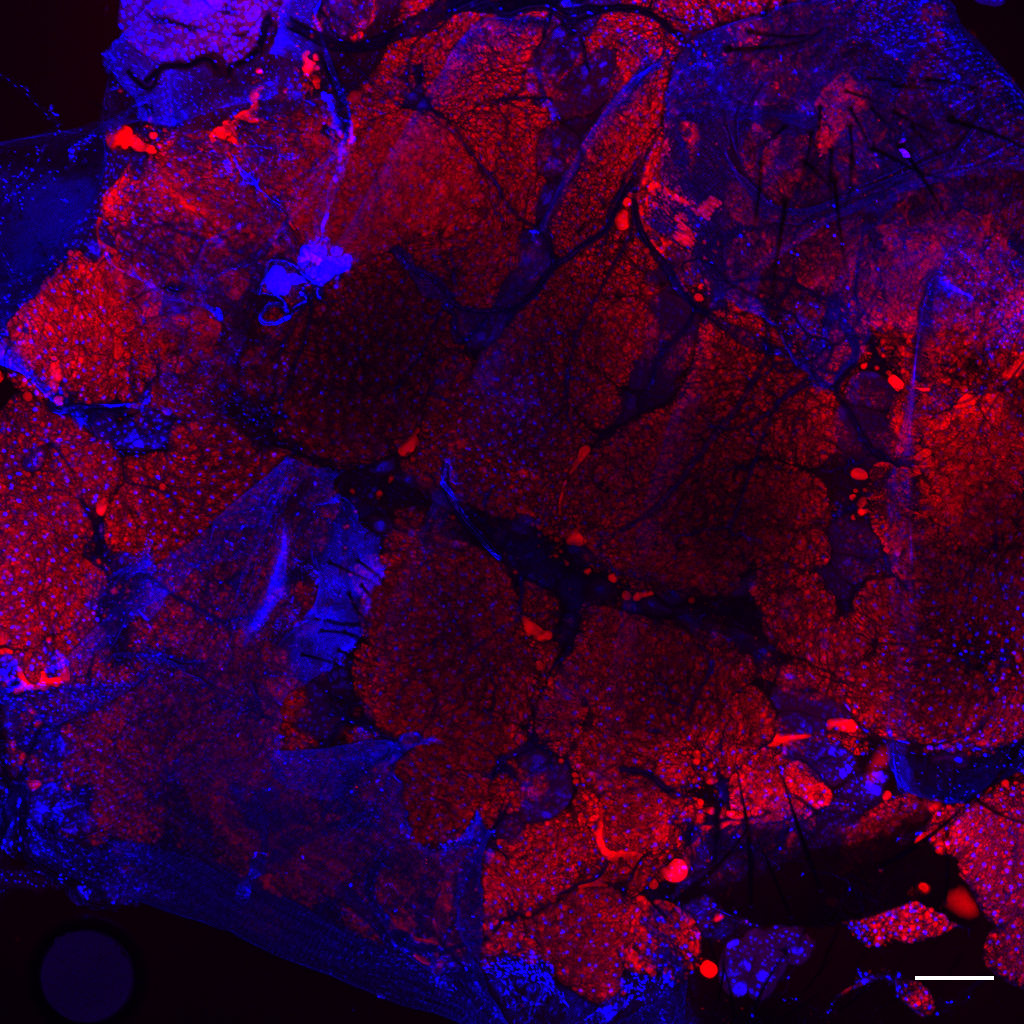

Supplement: Supplementary file 4 — Source data Fig. 2 [file 44319_2025_416_MOESM4_ESM.zip › 2E/MAX_LipidTOX Red 280919.lif - 75MA 10x -4.tif (RGB).tif]

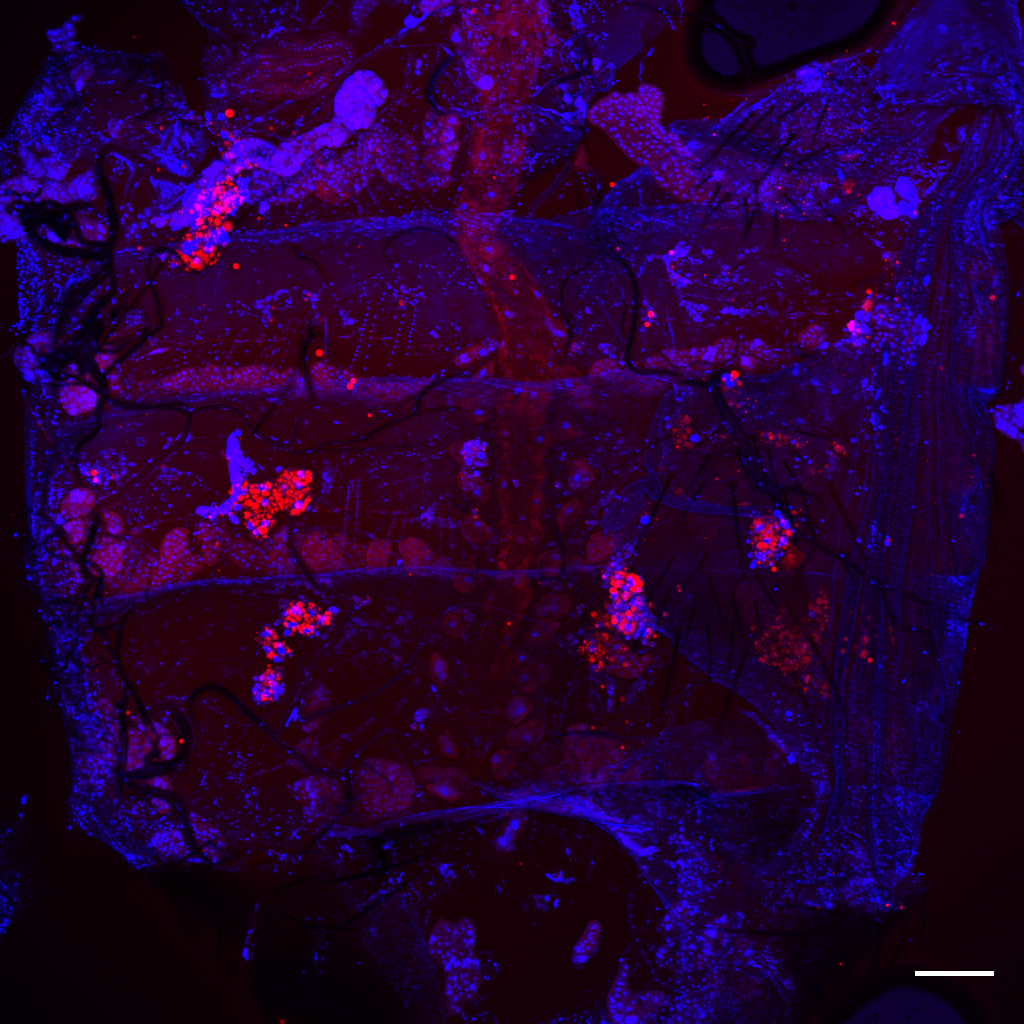

Supplement: Supplementary file 4 — Source data Fig. 2 [file 44319_2025_416_MOESM4_ESM.zip › 2E/MAX_LipidTOX Red 280919.lif - 75MD1A 10x -2.tif (RGB).tif]

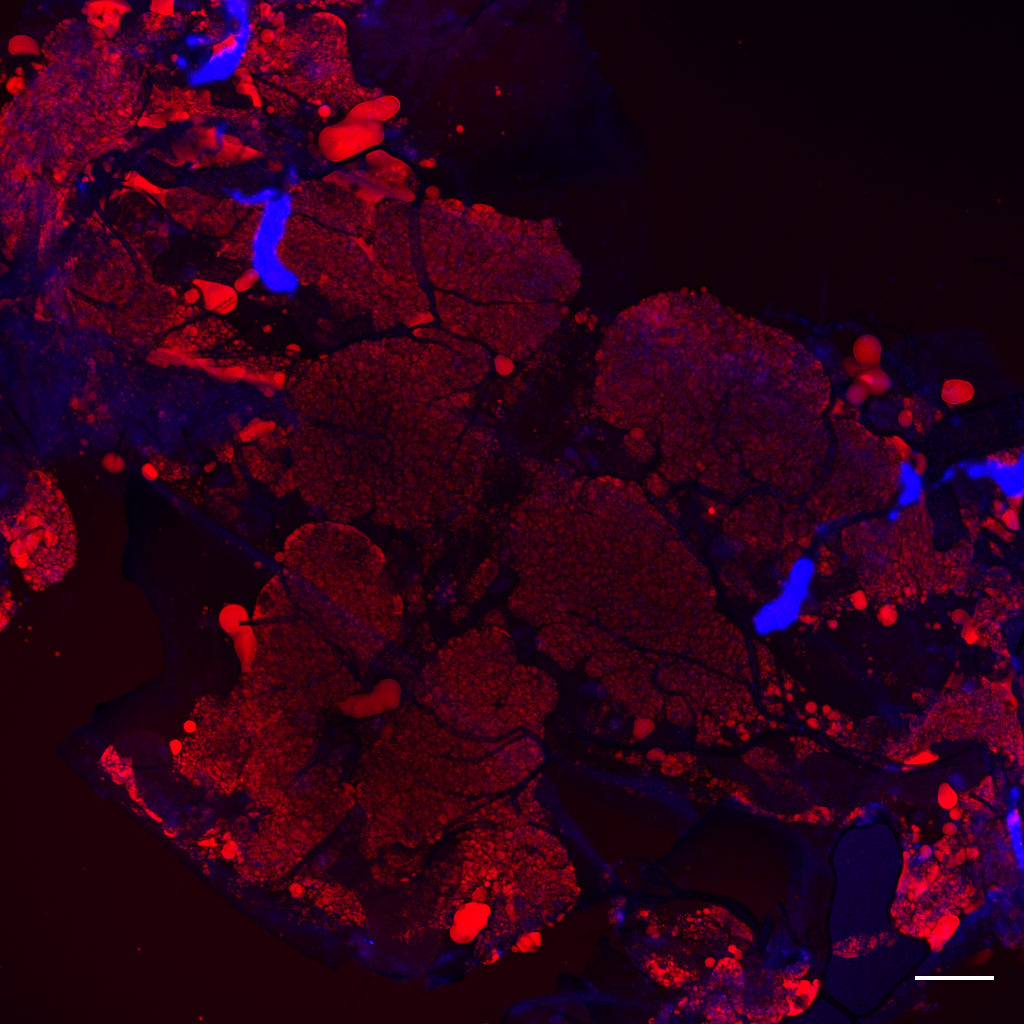

Supplement: Supplementary file 4 — Source data Fig. 2 [file 44319_2025_416_MOESM4_ESM.zip › 2E/MAX_LipidTOX Red 280919.lif - CMA 10x -4.tif (RGB).tif]

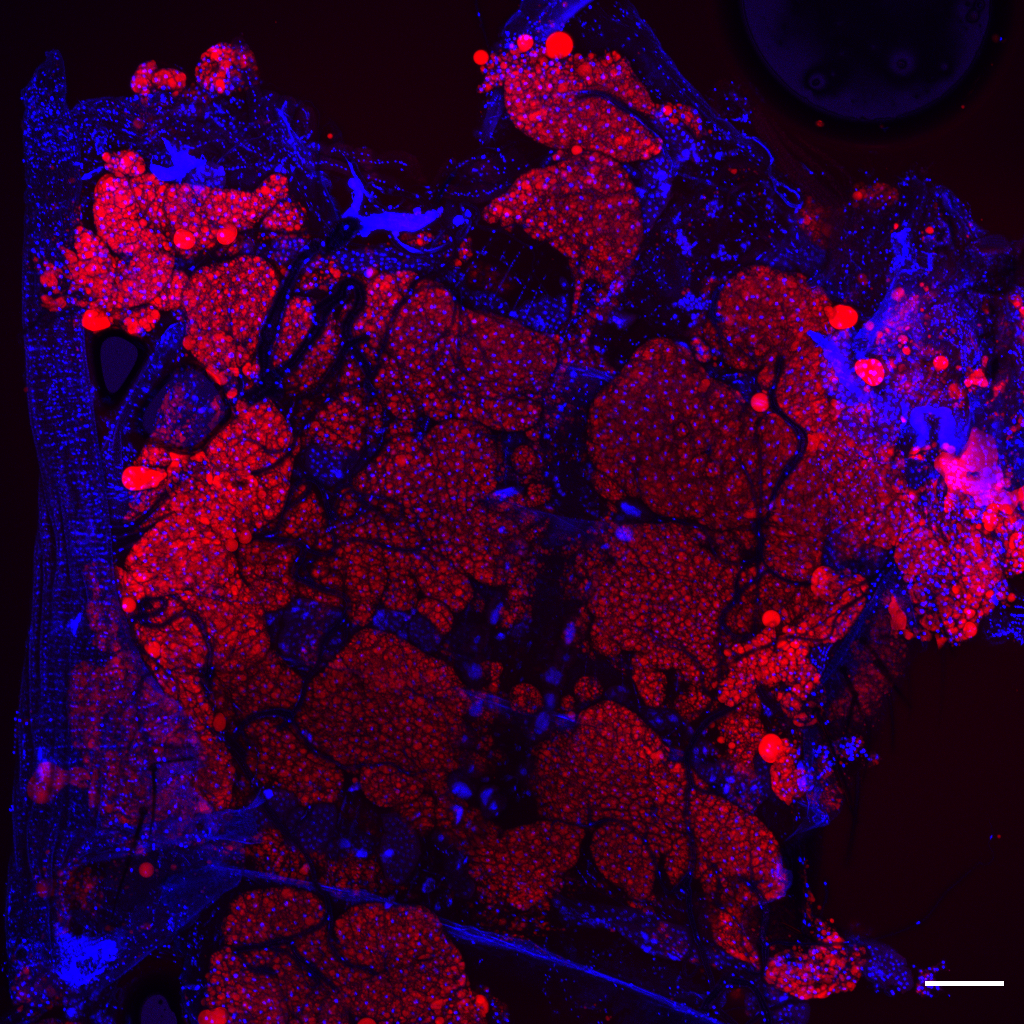

Supplement: Supplementary file 4 — Source data Fig. 2 [file 44319_2025_416_MOESM4_ESM.zip › 2E/MAX_LipidTOX Red 280919.lif - CMD1A 10x -1.tif (RGB).tif]
