# Supplementary material for: Developmental mitochondrial complex I activity determines lifespan
Source: EMBO Rep. 2025 Mar 17;26(8):1957–83. doi: 10.1038/s44319-025-00416-6 (PMC12019323; doi:10.1038/s44319-025-00416-6)
Supplement: Supplementary file 7 — Expanded View Figures [file 44319_2025_416_MOESM7_ESM.pdf]

Expanded View Figures

Figure EV1. Related to Figure 1.

(**A**) mRNA quantification of KD in ND-18 KD (left) and ND-75 KD (right) adult males. Kolmogorov–Smirnov test, $P = 0.100$ (ND-18 KD) & $P = 0.100$ (ND-75 KD), $n = 3$ independent samples per experimental group. (**B**) mRNA quantification of KD in ND-18 KD (left) and ND-75 KD (right) 3rd instar larvae in the presence (drug) or absence (vehicle) of the GS inducer, RU-486. *T* test, $P = 0.0003$ (ND-18 KD) & $P = 0.0009$ (ND-75 KD), $n = 4$ independent samples per experimental group. (**C**) Western blot analysis of CI core subunit, NDUFV2 (ND-24) in control males and males where ND-18 or ND-75 has been depleted from early development (D + A) or from adulthood only (A-only), beta-actin was used as a loading control. ANOVA with Tukey's multiple comparison test ($P = 0.0059$ (ND-18 KD) & $P = 0.0069$ (ND-75 KD)). $n = 3$–6 independent samples per experimental group. (**D**) Quantification of ND-18 (upper) and ND-75 (lower) mRNA levels in adult males exposed to the inducer from early development (D + A) or from adulthood (A-only). *T* test, $P = 0.2852$ (ND-18 KD) & $P = 0.5807$ (ND-75 KD), $n = 3$ independent samples per experimental group. (**E**) Survival of male control flies exposed to the inducer, RU-486, from early development (D + A) or from adulthood (A-only). Log-rank test, $P = 0.1120$, $n = 223$–262 individuals per experimental group. (**F**) Survival of female flies where CI subunit, ND-18, has been depleted from early development (D + A) or from adulthood (A-only). Log-rank test, $P < 0.001$, $n = 50$–58 flies per experimental group. (**G**) Survival of female flies where CI subunit, ND-75, has been depleted from early development (D + A) or from adulthood (A-only). Log-rank test, $P < 0.001$, $n = 55$–57 flies per experimental group. (**H**) Levels of CI-linked respiration in control adult males exposed to the inducer from early development (D + A) or from adulthood (A-only). *T* test, $P = 0.2045$, $n = 10$–11 independent samples per group. (**I**) % Eclosion of flies where depletion of CI subunit, ND-75, has been induced at distinct developmental stages. $n = 5$ independent samples per experimental group. (**J**) Survival of male flies where depletion of CI subunit, ND-75, has been induced at distinct developmental stages. $n = 92$–100 flies per experimental group. (**K**) % Eclosion of control male flies moved from 18 °C to 29 °C at distinct developmental stages. $n = 4$–5 independent samples per experimental group. (**L**) Survival of control male flies moved from 18 °C to 29 °C at distinct developmental stages. $n = 26$–72 flies per experimental group. In bar graphs, means are presented with ± SEM. Significance is indicated as follows: $^*P < 0.05$, $^{**}P < 0.01$, $^{***}P < 0.001$; ns = not significant.

© The Author(s)

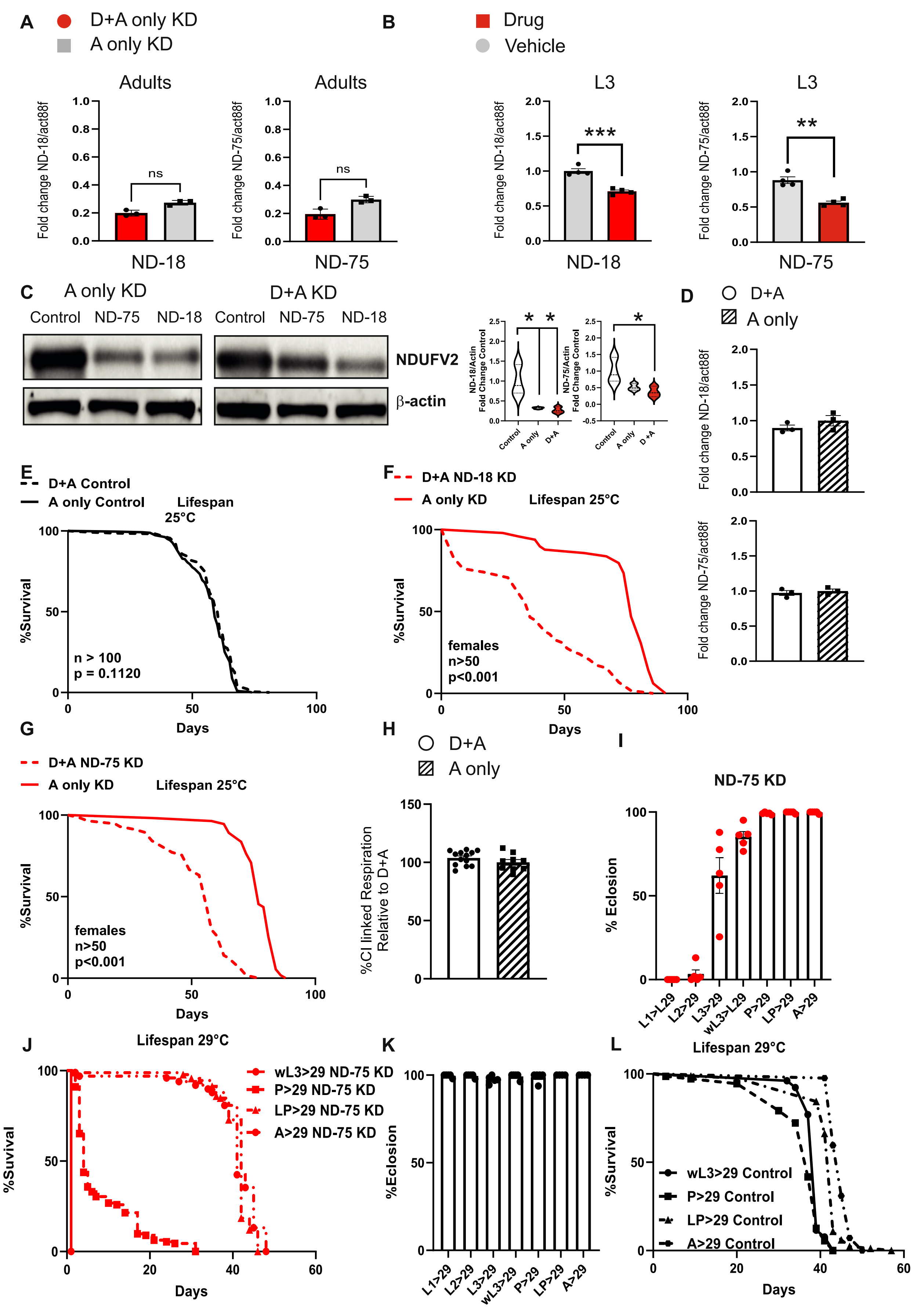
A
D+A only KD
A only KD
Adults
Fold change ND-18/act88f
ns
ND-18
Adults
Fold change ND-75/act88f
ns
ND-75
B
Drug
Vehicle
L3
Fold change ND-18/act88f
***
ND-18
L3
Fold change ND-75/act88f
**
ND-75
C
A only KD
D+A KD
Control ND-75 ND-18 Control ND-75 ND-18
NDUFV2
β-actin
ND-18/Actin Fold Change Control
ND-75/Actin Fold Change Control
Control A only D+A
D
D+A
A only
Fold change ND-18/act88f
Fold change ND-75/act88f
E
D+A Control
A only Control
Lifespan 25°C
%Survival
n > 100
p = 0.1120
Days
F
D+A ND-18 KD
A only KD
Lifespan 25°C
%Survival
females
n>50
p<0.001
Days
G
D+A ND-75 KD
A only KD
Lifespan 25°C
%Survival
females
n>50
p<0.001
Days
H
D+A
A only
%CI linked Respiration Relative to D+A
I
ND-75 KD
% Eclosion
L1>L29 L2>29 L3>29 wL3>L29 P>29 LP>29 A>29
J
Lifespan 29°C
%Suvival
wL3>29 ND-75 KD
P>29 ND-75 KD
LP>29 ND-75 KD
A>29 ND-75 KD
Days
K
%Eclosion
L1>29 L2>29 L3>29 wL3>29 P>29 LP>29 A>29
L
Lifespan 29°C
%Survival
wL3>29 Control
P>29 Control
LP>29 Control
A>29 Control
Days

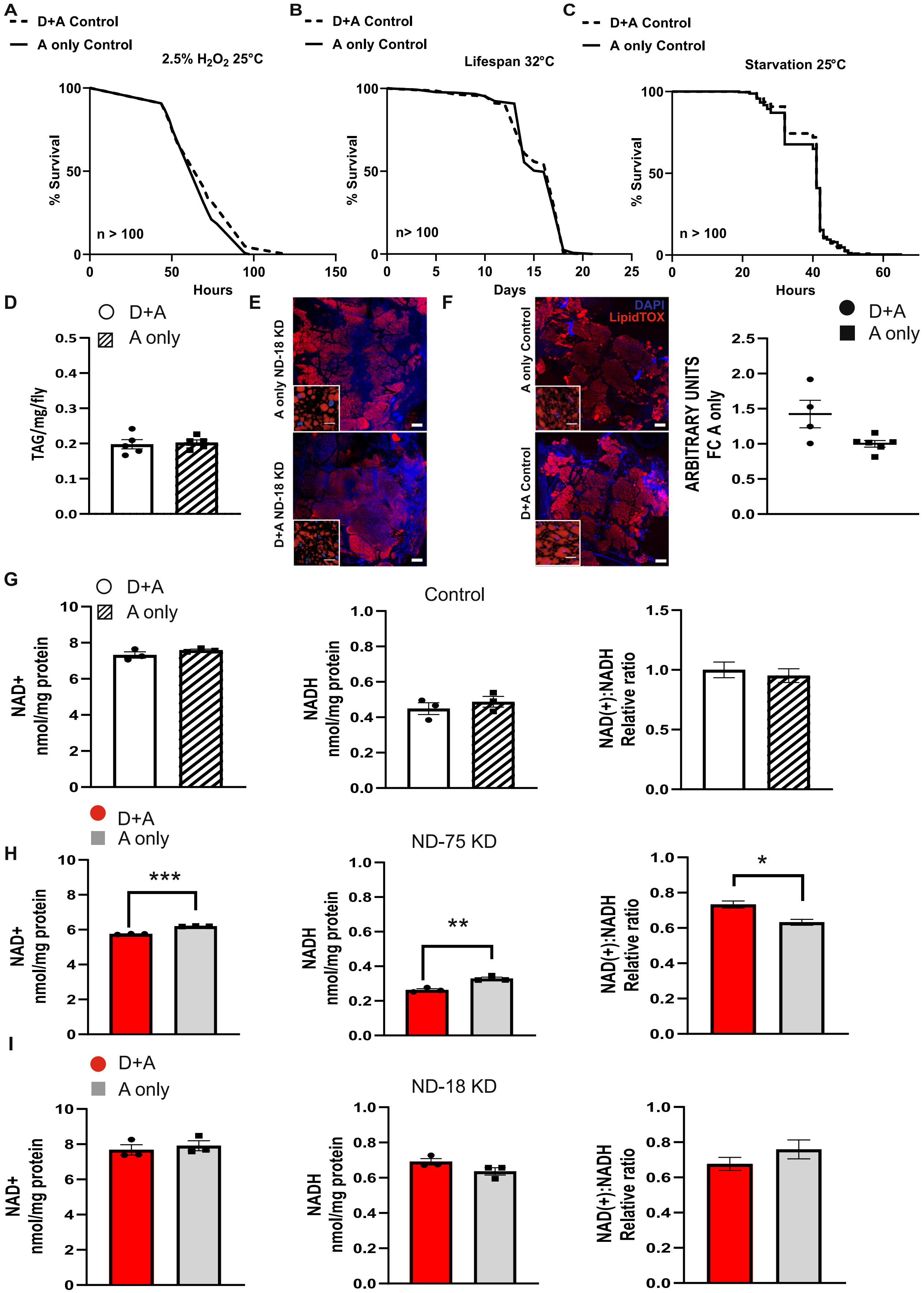
A
D+A Control
A only Control
2.5% H_2O_2 25°C
% Survival
n > 100
Hours
B
D+A Control
A only Control
Lifespan 32°C
% Survival
n> 100
Days
C
D+A Control
A only Control
Starvation 25°C
% Survival
n > 100
Hours
D
D+A
A only
TAG/mg/fly
E
A only ND-18 KD
D+A ND-18 KD
F
DAPI
LipidTOX
A only Control
D+A Control
D+A
A only
ARBITRARY UNITS
FC A only
G
D+A
A only
NAD+
nmol/mg protein
Control
NADH
nmol/mg protein
NAD(+):NADH
Relative ratio
D+A
A only
H
ND-75 KD
***
**
*
NAD+
nmol/mg protein
NADH
nmol/mg protein
NAD(+):NADH
Relative ratio
I
D+A
A only
ND-18 KD
NAD+
nmol/mg protein
NADH
nmol/mg protein
NAD(+):NADH
Relative ratio

© The Author(s)

Figure EV2. Related to Figure 2.

Survival under oxidative stress (**A**), Log-rank test, $P = 0.0979$, $n = 156$–159 individuals per group), thermal stress (**B**), Log-rank test, $P = 0.5462$, $n = 274$–281 individuals per group), and starvation (**C**), Log-rank test, $P = 0.8445$, $n = 250$–254 individuals per group) conditions of control male flies exposed to the inducer, RU-486 from early development (D+A) or from adulthood (A-only). (**D**) Quantification of triacylglyceride levels in control male flies exposed to the inducer, RU-486 from early development (D+A) or from adulthood (A-only). *T* test, $P = 0.7662$. $n = 5$ independent samples per experimental group. (**E**) Confocal imaging of fat bodies from male flies where ND-18 has been depleted from D + A or A-only stained with LipidTOX Red and DAPI, scale bar (90 µM), inset scale bar (10 µM). $n = 2$ independent samples per group. (**F**) Confocal imaging of fat bodies from male flies exposed to the inducer, RU-486 from early development (D+A) or from adulthood (A-only) stained with LipidTOX Red and DAPI, scale bar (90 µM), inset scale bar (10 µM). *T* test, $P = 0.1168$, $n = 4$–6 independent samples per experimental group. (**G**) Levels of NAD+ (*t* test, $P = 0.2269$), NADH (*t* test, $P = 0.4408$), and the relative ratio of NAD(+):NADH (*t* test, $P = 0.6101$) in control flies. $n = 3$ independent samples per group. (**H**) Levels of NAD+ (*t* test, $P < 0.001$), NADH (*t* test, $P = 0.0041$), and the relative ratio of NAD(+):NADH (*t* test, $P = 0.0179$) in ND-75 KD flies. $n = 3$ independent samples per group. (**I**) Levels of NAD+ (*t* test, $P = 0.5993$), NADH (*t* test, $P = 0.1191$), and the relative ratio of NAD(+):NADH (*t* test, $P = 0.2759$) in ND-18 KD flies. $n = 3$ independent samples per group. In bar graphs, means are presented with ± SEM. Significance is indicated as follows: $^*P < 0.05$, $^{**}P < 0.01$, $^{***}P < 0.001$; n.s. = not significant.

© The Author(s)

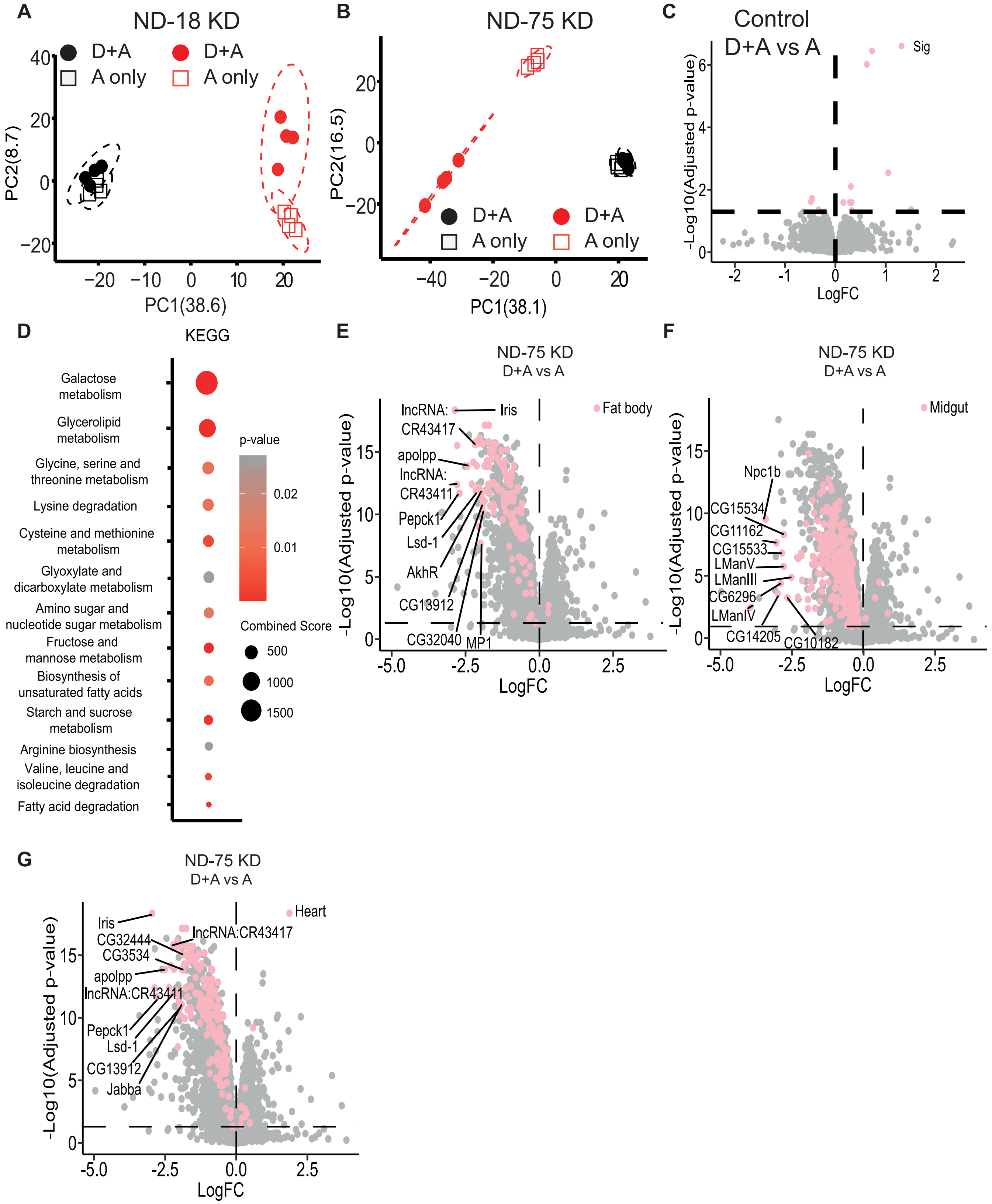
A
ND-18 KD
D+A
A only
D+A
A only
PC2(8.7)
PC1(38.6)
B
ND-75 KD
PC2(16.5)
PC1(38.1)
C
Control
D+A vs A
Sig
-Log10(Adjusted p-value)
LogFC
D
KEGG
Galactose metabolism
Glycerolipid metabolism
Glycine, serine and threonine metabolism
Lysine degradation
Cysteine and methionine metabolism
Glyoxylate and dicarboxylate metabolism
Amino sugar and nucleotide sugar metabolism
Fructose and mannose metabolism
Biosynthesis of unsaturated fatty acids
Starch and sucrose metabolism
Arginine biosynthesis
Valine, leucine and isoleucine degradation
Fatty acid degradation
p-value
Combined Score
E
ND-75 KD
D+A vs A
Fat body
lncRNA: CR43417
Iris
apolpp
lncRNA: CR43411
Pepck1
Lsd-1
AkhR
CG13912
CG32040
MP1
F
ND-75 KD
D+A vs A
Midgut
Npc1b
CG15534
CG11162
CG15533
LManV
LManIII
CG6296
LManIV
CG14205
CG10182
G
ND-75 KD
D+A vs A
Heart
Iris
CG32444
CG3534
apolpp
lncRNA:CR43411
lncRNA:CR43417
Pepck1
Lsd-1
CG13912
Jabba

Figure EV3. Related to Figure 3.

(**A**) PCA analysis of controls and ND-18 KD D + A and A-only conditions. $n = 4$ independent samples per group. (**B**) PCA analysis of controls and ND-75 KD D + A and A-only conditions. $n = 4$ independent samples per group. (**C**) Volcano plot of genes significantly differentially expressed between control D + A and control A. FDR, $P < 0.05$, $n = 4$ independent samples per group. (**D**) KEGG Enrichment Analysis (FlyEnricher) performed on those genes significantly altered in both ND-18 D + A vs A-only and ND-75 D + A vs A-only reveals significant enrichment in the following classifications. FDR, $P < 0.05$, $n = 4$ independent samples per group. (**E-G**) Volcano plots of genes significantly differentially expressed between ND-75 D + A and ND-75 A-only (gray), highlighted in pink, are those genes with highly enriched expression in the fat body (**G**), midgut (**H**) and heart (**I**). FDR, $P < 0.05$, $n = 4$ independent samples per group.

© The Author(s)

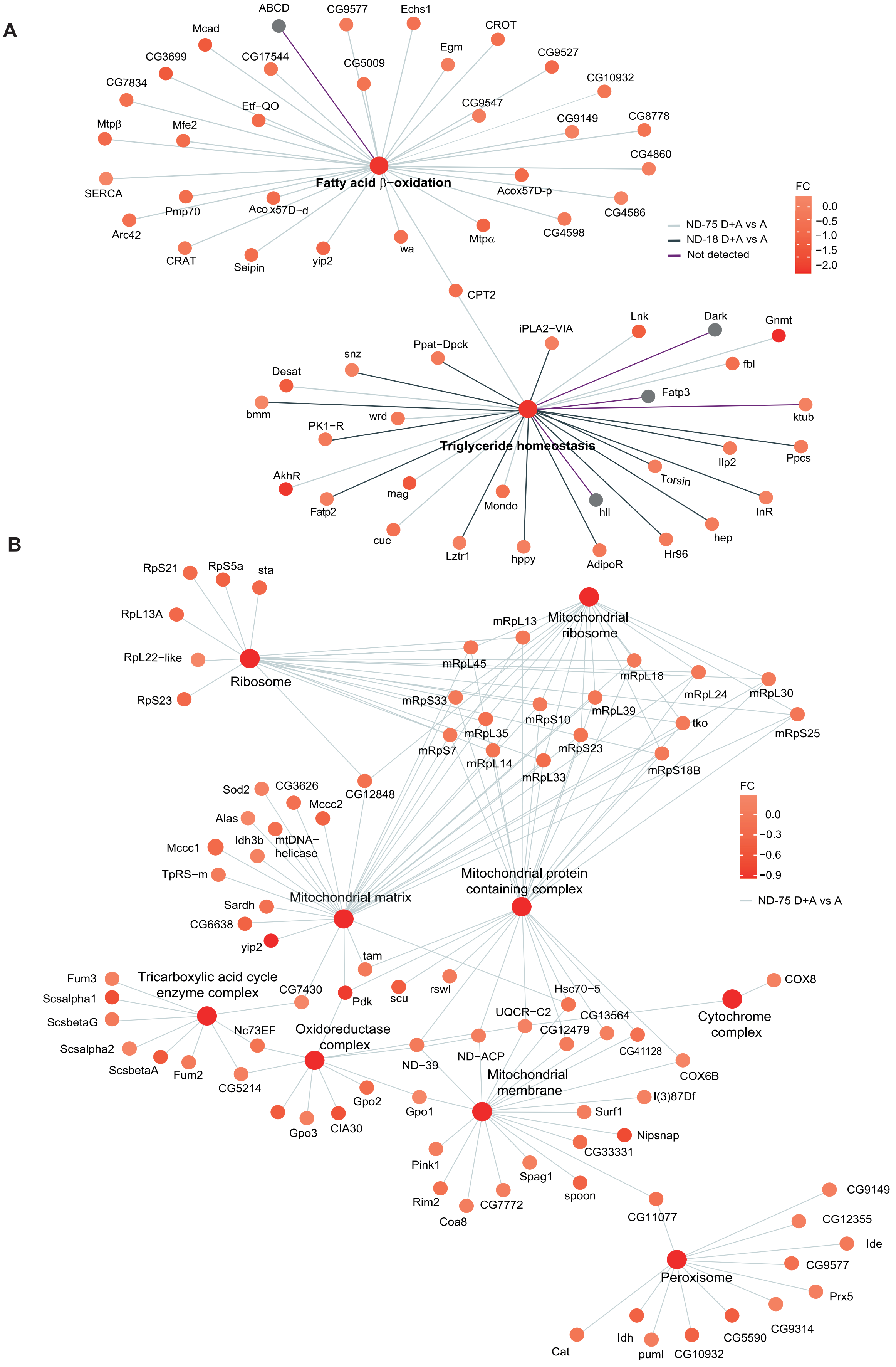
A
ABCD
CG9577
Echs1
CROT
Mcad
Egm
CG9527
CG3699
CG17544
CG5009
CG10932
CG7834
Etf-QO
CG9547
CG8778
Mtpβ
Mfe2
CG9149
CG4860
SERCA
Fatty acid β−oxidation
Acox57D-p
Pmp70
Aco x57D−d
CG4586
Arc42
CG4598
CRAT
Seipin
yip2
wa
Mtpα
CPT2
FC
0.0
−0.5
−1.0
−1.5
−2.0
ND-75 D+A vs A
ND-18 D+A vs A
Not detected
Lnk
Dark
Gnmt
iPLA2−VIA
Ppat−Dpck
fbl
snz
Desat
Fatp3
bmm
ktub
wrd
PK1−R
Triglyceride homeostasis
Ilp2
Ppcs
AkhR
Torsin
mag
Mondo
InR
Fatp2
hll
hep
cue
Lztr1
hppy
AdipoR
Hr96
B
RpS21
RpS5a
sta
Mitochondrial ribosome
RpL13A
mRpL13
RpL22−like
mRpL45
Ribosome
mRpL18
mRpL24
mRpL30
RpS23
mRpS33
mRpL39
mRpS10
tko
mRpS25
mRpL35
mRpS23
mRpS7
mRpL14
mRpS18B
mRpL33
Sod2
CG3626
CG12848
Mccc2
FC
0.0
−0.3
−0.6
−0.9
Alas
Idh3b
mtDNA−helicase
Mccc1
TpRS−m
Mitochondrial protein containing complex
Mitochondrial matrix
ND-75 D+A vs A
Sardh
CG6638
yip2
tam
Fum3
Tricarboxylic acid cycle enzyme complex
CG7430
rswl
Hsc70−5
COX8
Scsalpha1
Pdk
scu
UQCR−C2
CG13564
Cytochrome complex
ScsbetaG
Nc73EF
Oxidoreductase complex
CG12479
Scsalpha2
CG41128
ND−39
ND−ACP
ScsbetaA
Fum2
CG5214
Mitochondrial membrane
COX6B
Gpo2
l(3)87Df
Gpo1
Surf1
Gpo3
CIA30
Nipsnap
CG33331
Pink1
Spag1
Rim2
CG7772
spoon
Coa8
CG11077
CG9149
CG12355
Ide
CG9577
Peroxisome
Prx5
CG9314
Cat
Idh
puml
CG10932
CG5590

 © The Author(s)

Figure EV4. Related to Figure 3.

(**A**, **B**) Clustergrams of the indicated GO terms expressing the differential expression (FC) of genes associated with this term. All FCs are from ND-75 KD D + A vs A-only comparison unless otherwise indicated by a dark gray line (ND-18 KD D + A vs A-only) or a purple line (not detected in our study). $n = 4$ independent samples per group.

Figure EV5

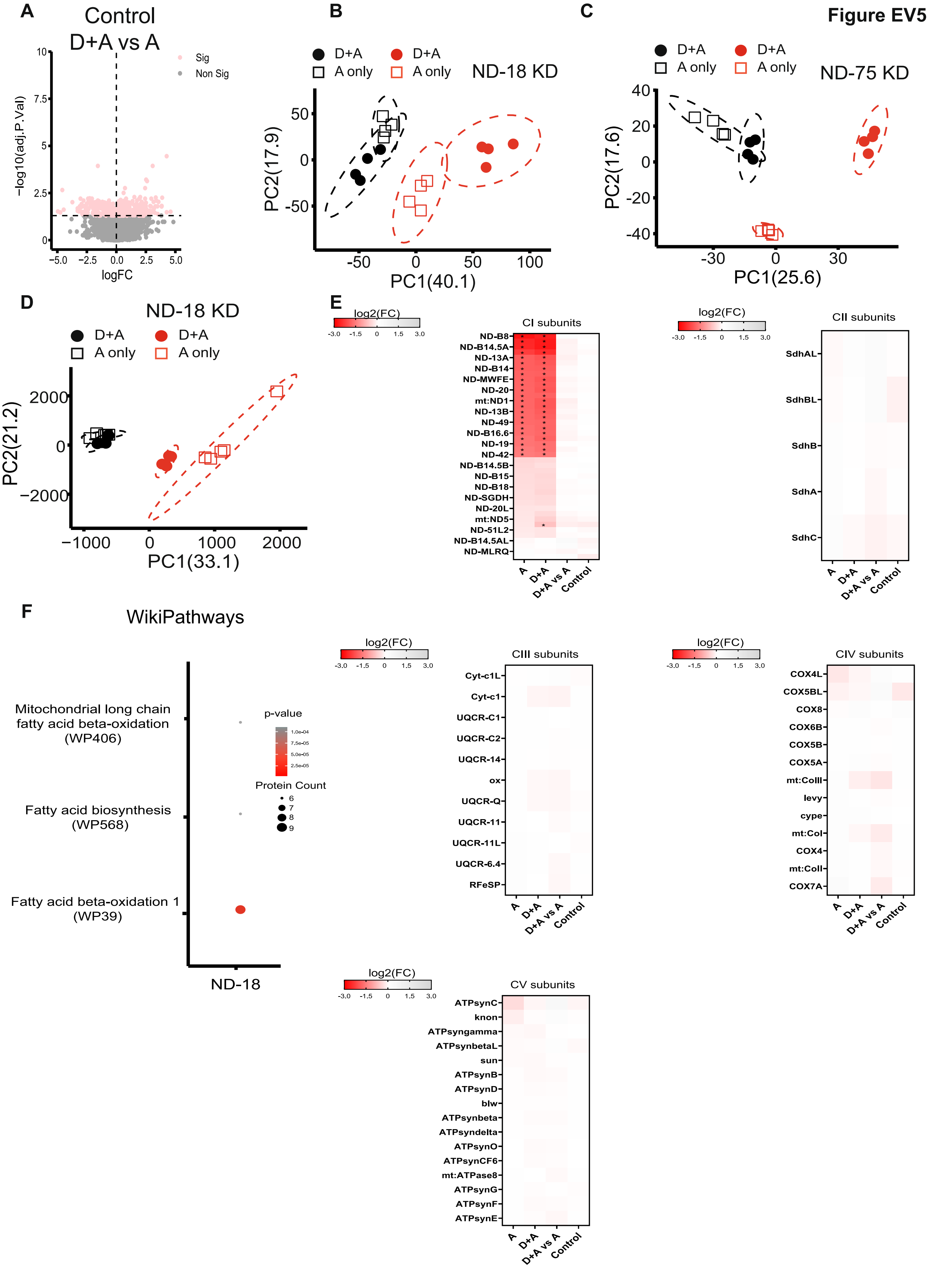

 © The Author(s)

Figure EV5. Related to Figure 4.

(**A**) Volcano plot of metabolites with significantly different abundance between control D + A and control A-only. FDR, $P < 0.05$, $n = 4$ independent samples per group. (**B**, **C**) PCA analysis of metabolomics data from controls and ND-18 KD (**B**) and controls and ND-75 KD (**C**) D + A vs A-only conditions. $n = 4$ independent samples per group. (**D**) PCA analysis of proteomics data from control and ND-18 KD D + A vs A-only conditions. $n = 5$ independent samples per group. (**E**) Heat maps depicting the expression of OXPHOS subunits (Complexes I–V). Asterisks (*) indicate significant differences in protein expression, FDR, $P < 0.05$. The comparisons are: (**A**) ND-18 A *versus* control A, (D + A) ND-18 D + A *versus* control D + A, (D + A vs A) ND-18 D + A *versus* ND-18 A, and (Control) control D + A versus control A. $n = 5$ independent samples per group. (**F**) Dot plot showing the most significant pathways according to STRING wikiPathways altered at the protein level in ND-18 KD D + A flies. FDR, $P < 0.05$. $n = 5$ independent samples per group.

© The Author(s)
